# Body surface area is a novel predictor for surgical complications following video-assisted thoracoscopic surgery for lung adenocarcinoma: a retrospective cohort study

**DOI:** 10.1186/s12893-017-0264-4

**Published:** 2017-06-12

**Authors:** Shuangjiang Li, Kun Zhou, Heng Du, Cheng Shen, Yongjiang Li, Guowei Che

**Affiliations:** 1 Department of Thoracic Surgery, West China Hospital, Sichuan University, Chengdu, China; 2 Department of Oncology, West China Hospital, Sichuan University, Chengdu, China

**Keywords:** Body surface area, Video-assisted thoracoscopic surgery, Complications, Prediction

## Abstract

**Background:**

Body surface area (BSA) is a biometric unit to measure the body size. Its clinical significance in video-assisted thoracoscopic surgery (VATS) was rarely understood. We aimed to estimate the predictive value of BSA for surgical complications following VATS anatomical resections for lung adenocarcinoma (LAC).

**Methods:**

A single-center retrospective analysis was performed on the consecutive patients between July 2014 and January 2016 in our institution. The differences in mean BSA values were evaluated between groups of patients classified by the development of postoperative surgical complications (PSCs), overall morbidity and cardiopulmonary complications, respectively. Receiver operating characteristic (ROC) analysis was performed to determine a threshold value of BSA on prediction of PSC occurrence. A multivariate logistic-regression model involving this optimal cut-off value and other significant parameters was established to identify the predictors for PSCs.

**Results:**

During the study period, a total of 442 patients undergoing VATS anatomical resections for LAC were enrolled in this study. There were 135 patients developed with one or more complications (rate = 30.5%). PSCs occupied the largest percentages of all these complications (*n* = 81, rate = 18.3%). The mean BSA in PSC group was significantly higher than that in non-PSC group (1.76 ± 0.15 m^2^ vs 1.71 ± 0.16 m^2^; *P* = 0.016). No difference was found in mean BSA values between groups classified by any other complication. The ROC analysis determined a BSA value of 1.68 m^2^ to be the threshold value with the maximum joint sensitivity of 72.8% and specificity of 48.5%. Compared to patients with BSA ≤ 1.68 m^2^, patients with BSA > 1.68 m^2^ had significantly higher incidences of prolonged air leak (*P* = 0.006) and chylothorax (*P* = 0.004). Further multivariate logistic-regression analysis indicated that BSA > 1.68 m^2^ could be an independent risk factor for PSCs (odds ratio: 2.03; *P* = 0.025).

**Conclusions:**

BSA is an excellent categorical predictor for surgical complications following VATS anatomical resections for LAC. It may be considered when informing patients about surgical risks and selecting cases in the early learning curve. Large-scale and multi-institutional studies are expected to confirm and modify our findings in the future.

**Electronic supplementary material:**

The online version of this article (doi:10.1186/s12893-017-0264-4) contains supplementary material, which is available to authorized users.

## Background

### Rationale

Lung cancer is the worldwide leading cause of malignancy-related deaths and remains the most prevalent cancer in both developed and developing countries [1]. Non-small cell lung cancer (NSCLC) accounts for approximately 85% of all lung cancer cases, with a poor 5-year survival rate of less than 15% [2]. Lung adenocarcinoma (LAC) is the most common histological subtypes of primary NSCLC and its incidence continues to grow in recent years [3]. Advances in radiographic techniques and the prevalent practice of cancer screening have increased the opportunity for early detection and intervention of LAC [4].

Nowadays, radical surgery is still regarded as the optimal therapeutic option for early-stage LAC, which leads to a significantly improved 5-year survival rate reaching up to 73% [4]. Since the 1990s, video-assisted thoracoscopic surgery (VATS) emerges as a minimally invasive procedure to gain access to the chest cavity and has been widely utilized in the modern surgical modality for early-stage LAC. The VATS has showed more advantages offered to patients than conventional thoracotomy, especially in pain and stress control, cosmetic wounds and preservation of pulmonary functions [5, 6].

However, despite advances in VATS techniques and perioperative care, postoperative complication rates still remain as high as 24.9–36.3% [6–8]. Minor complications caused by surgical procedures, such as prolonged air leak (PAL) and pneumothorax, can significantly prolong the hospital stay and hinder postoperative recovery. Several fatal complications related to surgical procedures, such as chylothorax and bronchial fistula, can even lead to a high mortality rate up to 50% [9, 10]. Therefore, it is essential to identify which candidates are considered at high risks of postoperative surgical complications (PSCs).

Body surface area (BSA) has been widely used as a biometric unit for the normalization of physiological variables and determination of appropriate drug dosages during chemotherapy [11]. However, its clinical significance in the surgical specialties was rarely reported in previous studies [12]. The relationships between BSA and surgical outcomes of VATS are still inadequately understood because of the scarcity of sufficient evidence [13].

### Objectives

Given above concerns, we aimed to estimate the value of BSA as a predictor for PSCs in patients undergoing VATS anatomical resections for LAC by performing a retrospective analysis.

## Methods

### Study designs

This study was a single-center retrospective analysis conducted on the data derived from a prospectively-maintained dataset with their medical records in our institution. It was written in compliance with the Strengthening the Reporting of Observational Studies in Epidemiology (STROBE) statement [14].

### Study protocol

Our study protocol was approved by the Regional Ethics Committee of Sichuan University West China Hospital.

### Participant selection

#### Settings

We retrospectively analyzed the clinical data of patients who underwent VATS anatomical resections for operable LAC from July 2014 to January 2016 in our unit. We carefully reviewed the medical records and collected all available data of patients’ characteristics and surgical outcomes for further analysis.

#### Eligibility criteria

First, the target disease was operable primary LAC. Squamous cell carcinoma and other subtypes of NSCLC would not be considered.

Second, elective anatomical resections operated by VATS procedures, including single lobectomy, bi-lobectomy and segmentectomy, were included in this study. Pneumonectomy, wedge and sleeve resections were excluded.

Third, patients who were unable to finish the entire clinical pathway due to various reasons were not included.

Fourth, patients with loss of details on evaluated variables or surgical outcomes could not be included.

#### Follow-up

The endpoints of our study belong to in-hospital outcomes. Thus, a follow-up would be provided for each patient until 30 days after surgery or death in hospital.

### Outcome measures and definitions

We recorded and defined the following estimated variables and outcome data.

#### Preoperative variables

Patient baseline information included the age, gender, body mass index (BMI, categorized according to the World Health Organization definitions), BSA and smoking history (formal/current/never-smoker).

Taking account of the ethnic differences between Chinese and Western peoples, we calculated the baseline BSA in this study according to the following formula for contemporary Chinese subjects [15]: BSA (m^2^) = [0.0061 × height (cm) + 0.0124 × weight (kg) – 0.0099].

Major underlying comorbidities included the chronic obstructive pulmonary disease (COPD), tuberculosis, preoperative respiratory infection (PRI), hypertension, diabetes mellitus, coronary heart disease, severe liver diseases, renal insufficiency, previous malignancy and steroid use. We defined PRI as the presence of one or more of the following infectious conditions: obstructive pneumonia, aspiration pneumonia, bronchiectasis, lung abscess and respiratory bacterial/fungal infections. The definitions for other above comorbidities are summarized in Table S1 (see the Additional file 1).

A multi-disciplinary team meeting would discuss the combined treatment modalities before surgery if necessary. Neoadjuvant therapy was determined as a cisplatin/paclitaxel-based chemo-radiotherapy in accordance with the National Comprehensive Cancer Network Guidelines: China Editions.

#### Intraoperative variables

Intraoperative parameters included in the comparable analysis are presented as follows: tumor location, extent of anatomical resection (single lobectomy/bi-lobectomy/segmentectomy), degrees of pleural adhesion (none/light/moderate/severe), pleural invasion (none/visceral/parietal) and pulmonary fissure completeness (complete/incomplete).

#### Pathological variables

We evaluated the following general pathological parameters, including the differentiation degrees (low/moderate/high), tumor invasion (T status), lymph node metastasis (N status) and TNM stages, which were defined according to the Union for International Cancer Control seventh edition.

#### Primary and secondary outcomes

The primary outcome of interest was PSCs, which included the PAL (>5 days), pneumothorax, chylothorax, subcutaneous emphysema, wound infection, hemothorax and bronchial fistula.

The secondary outcomes of interest were overall morbidity, pulmonary complications and cardiovascular complications. Overall morbidity was defined by presence of any individual postoperative complication. Pulmonary complications consisted of the pneumonia, atelectasis, pulmonary embolism, acute respiratory distress syndrome and pleural effusion. Cardiovascular complications included the atrial arrhythmia, ventricular arrhythmia, myocardial infarction and cerebrovascular events.

All of above complications were judged in accordance with the Society of Thoracic Surgeons and the European Society of Thoracic Surgeons joint definitions [16].

### Grouping criterion

Firstly, patients were divided into the group of patients with PSCs and the group of patients without PSCs. Then, we compared the patient characteristics between these two groups, in order to initially identify the clinicopathological parameters that were significantly associated with the development of PSCs.

Secondly, we performed a receiver operating characteristic (ROC) analysis to determine a threshold value of BSA that had the discriminatory ability to predict the occurrence of PSCs. Then, we compared the incidences of individual PSCs between patients with BSA above the optimal cut-off value and patients with BSA below the optimal cut-off value. This threshold value of BSA would be finally included in the multivariate logistic-regression model to stratify the patients at high risk of PSCs.

### Surgical procedure and perioperative care

All of enrolled patients were operated by a VATS procedure using the single-direction thoracoscopic technique that was described by Liu et al. [17] through a three-portal access. A systematic mediastinal lymphadenectomy was performed on all of these patients, and a stapler line reinforcement was also utilized on their bronchial stumps.

Every patient was managed in compliance with our standardized clinical pathway, including comprehensive routine assessments, antibiotic prophylaxis, pulmonary rehabilitation and physiotherapy before surgery [18]. All surgical patients received intravenous patient-controlled analgesia for postoperative pain control. A chest tube was placed on the suction device (−20 cm H_2_O) at the end of operation, and later either alternated or removed the suction according to institutional policies. The degrees of lung expansion was stratified by a chest X-ray on postoperative day 1. The chest tube would be removed when the pleural drainage remained <200 ml in 24 h and the air leak cessation was detected in chest drainage unit. The analgesic management would be stopped once the chest tube was removed.

### Statistical analysis

We used the SPSS 22.0 software to accomplish the following statistical analyses.

The continuous data was presented as the mean with standard deviation (mean ± SD), while the dichotomous data was expressed as the patient number with their percentages.

The univariate analysis utilized the Pearson’s chi-squared test or Fisher’s exact test to compare the dichotomous data, and the Student’s *t*-test to compare the mean values of continuous data.

The ROC analysis was conducted to evaluate the discriminative power of BSA on predictions for PSCs. The area under curve (AUC) with its 95% confidence interval (CI) was also calculated.

Finally, we performed a multivariate logistic-regression analysis to identify the independent risk factors for PSCs. The significant variables with a univariate *P* value <0.05 would be included into the multivariate logistic-regression model. Odds ratio (OR) with the corresponding 95% CI was also extrapolated.

The statistical significance would be revealed in both univariate and multivariate analysis when *P* value <0.05.

## Results

### Basic information and outcomes

#### Patient characteristics

Between July 2014 and January 2016, a total of 442 patients who underwent VATS anatomical resections for LAC met the inclusion criteria and were enrolled in this study.

**Fig. 1:**
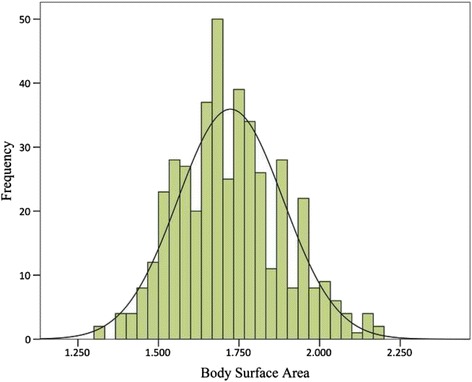
Frequency distribution histogram of body surface area

Patient baseline characteristics were shown in Table 1. This cohort consisted of 232 male (ratio = 52.5%) and 210 female patients (ratio = 47.5%), with a mean age of 62.84 ± 8.07 years (ranged 38–82 years). A frequency distribution histogram of BSA is shown as Fig. 1. The mean BMI and BSA of the entire cohort were 23.45 ± 3.01 kg/m^2^ (ranged 16.23–31.64 kg/m^2^) and 1.72 ± 0.16 m^2^ (ranged 1.31–2.18 m^2^), respectively. One hundred and sixty-seven patients were active smokers (ratio = 37.8%), and 328 patients had preoperative comorbidities (ratio = 74.2%), as shown in Table 1. There were 31 patients received neoadjuvant chemotherapy before surgery (ratio = 7.0%), while 122 patients were treated with adjuvant chemotherapy followed by surgery (ratio = 27.6%).

**Table 1:** Patient characteristics

Characteristics	Total (*N* = 442)	Surgical complications		*P* value
		Yes (*N* = 81)	No (*N* = 361)	
Basic information				
Mean age, years (SD)	62.84 ± 8.07	64.47 ± 7.18	62.48 ± 8.22	0.044
Age categorization				
Age ≤ 65 years	277 (62.7%)	48 (59.3%)	229 (63.4%)	0.48
Age > 65 years	165 (37.3%)	33 (40.7%)	132 (36.6%)	
Gender (Male, %)	232 (52.5%)	62 (76.5%)	170 (47.1%)	<0.001
Mean BMI, kg/m^2^ (SD)	23.45 ± 3.01	23.80 ± 3.10	23.37 ± 2.98	0.25
BMI categorization				
< 18.5 kg/m^2^	21 (4.8%)	2 (2.5%)	19 (5.3%)	0.51
18.5 to 25.0 kg/m^2^	300 (67.9%)	53 (65.4%)	247 (68.4%)	
> 25.0 to <30.0 kg/m^2^	114 (25.8%)	24 (29.6%)	90 (24.9%)	
≥ 30 kg/m^2^	7 (1.6%)	2 (2.5%)	5 (1.4%)	
Mean BSA, m^2^ (SD)	1.72 ± 0.16	1.76 ± 0.15	1.71 ± 0.16	0.016
Median BSA, m^2^ (Range)	1.71 (1.31–2.18)	1.76 (1.38–2.15)	1.69 (1.31–2.18)	
Smoking history	167 (37.8%)	46 (56.8%)	121 (33.5%)	<0.001
Preoperative comorbidities				
COPD	87 (19.7%)	25 (30.9%)	62 (17.2%)	0.005
Tuberculosis	44 (10.0%)	6 (7.4%)	38 (10.5%)	0.40
Preoperative respiratory infection	38 (8.6%)	15 (18.5%)	23 (6.4%)	<0.001
Hypertension	148 (33.5%)	38 (46.9%)	110 (30.5%)	0.005
Diabetes mellitus	47 (10.6%)	5 (6.2%)	42 (11.6%)	0.15
Coronary heart disease	45 (10.2%)	13 (16.0%)	32 (8.9%)	0.053
Renal insufficiency	32 (7.2%)	8 (9.9%)	24 (6.6%)	0.31
Severe liver diseases	53 (12.0%)	9 (11.1%)	44 (12.2%)	0.79
Previous malignancy	32 (7.2%)	8 (9.9%)	24 (6.6%)	0.31
Steroid use	23 (5.2%)	6 (7.4%)	17 (4.7%)	0.48
Combined treatment modalities				
Neoadjuvant therapy	31 (7.0%)	7 (8.6%)	24 (6.6%)	0.53
Adjuvant chemotherapy	122 (27.6%)	26 (32.1%)	96 (26.6%)	0.32
Intraoperative parameters				
Tumor location				
Right upper lobe	159 (36.0%)	22 (27.2%)	137 (38.0%)	0.24
Left upper lobe	100 (22.6%)	17 (21.0%)	83 (23.0%)	
Right lower lobe	78 (17.6%)	19 (23.5%)	59 (16.3%)	
Left lower lobe	61 (13.8%)	12 (14.8%)	49 (13.6%)	
Right middle lobe	44 (10.0%)	11 (13.6%)	33 (9.1%)	
Extent of surgery				
Single lobectomy	369 (83.5%)	74 (91.4%)	295 (81.7%)	0.011
Bi-lobectomy	4 (0.9%)	2 (2.5%)	2 (0.6%)	
Segmentectomy	69 (15.6%)	5 (6.2%)	64 (17.7%)	
Pleural invasion				
None	206 (46.6%)	34 (42.0%)	172 (47.6%)	0.65
Visceral	207 (46.8%)	41 (50.6%)	166 (46.0%)	
Parietal	29 (6.6%)	6 (7.4%)	23 (6.4%)	
Pleural adhesion				
None	199 (45.0%)	26 (32.1%)	173 (47.9%)	0.007
Light	132 (29.9%)	25 (30.9%)	107 (29.6%)	
Moderate	71 (16.1%)	16 (19.8%)	55 (15.2%)	
Severe/atresia	40 (9.0%)	14 (17.3%)	26 (7.2%)	
Pulmonary fissure status				
Complete fissures	300 (67.9%)	50 (61.7%)	250 (69.3%)	0.19
Incomplete fissures	142 (32.1%)	31 (38.3%)	111 (30.7%)	
Pathological parameters				
Differentiation degree				
Low	41 (9.3%)	12 (14.8%)	29 (8.0%)	0.091
Moderate/high	401 (90.7%)	69 (85.2%)	332 (92.0%)	
Tumor invasion (T status)				
T_1–2_	425 (96.2%)	74 (91.4%)	351 (97.2%)	0.030
T_3–4_	17 (3.8%)	7 (8.6%)	10 (2.8%)	
Lymph node metastasis (N status)				
N_1–2_	78 (17.6%)	17 (21.0%)	61 (16.9%)	0.38
N_0_	364 (82.4%)	64 (79.0%)	300 (83.1%)	
TNM stage				
I	346 (78.3%)	58 (71.6%)	288 (79.8%)	0.15
II	54 (12.3%)	16 (19.7%)	38 (10.5%)	
IIIa	42 (9.5%)	7 (8.6%)	35 (9.7%)	

*BMI* body mass index, *BSA* body surface area, *COPD* chronic obstructive pulmonary disease, *SD* standard deviation

The great majority of patients were diagnosed with stage I-II LAC (*n* = 400, ratio = 90.5%). Lymph node metastasis was confirmed by pathological criteria in 78 patients (ratio = 17.6%). A single lobectomy was performed on 369 patients (ratio = 83.5%), followed by segmentectomy on 69 patients (ratio = 15.6%) and bi-lobectomy on 4 patients (ratio = 0.9%). The LAC located in right upper lobe (*n* = 159) accounted for the largest proportion of all the enrolled patients, with a rate of 36.0%. The other clinicopathological parameters are also summarized in Table 1.

#### Surgical outcomes

There were 135 patients developed with one or more complications, with an overall morbidity rate of 30.5%. PSCs occupied the largest percentages of all types of complications, which were developed in 81 patients (ratio = 18.3%), followed by pulmonary complications in 71 patients (ratio = 16.1%) and cardiovascular complications in 13 patients (ratio = 2.9%), as shown in Table 2. There was no death during the hospitalization period.

**Table 2:** Postoperative complications

Outcomes	Total (*N* = 442)	Mean BSA value (SD)	*P* value
Overall morbidity			
Yes	135 (30.5%)	1.73 ± 0.16	0.44
No	307 (69.5%)	1.72 ± 0.17	
Surgical complications			
Yes	81 (18.3%)	1.76 ± 0.15	0.016
No	361 (81.7%)	1.71 ± 0.16	
Pulmonary complications			
Yes	71 (16.1%)	1.72 ± 0.16	0.99
No	371 (83.9%)	1.72 ± 0.16	
Cardiovascular complications			
Yes	13 (2.9%)	1.67 ± 0.12	0.19
No	429 (97.1%)	1.73 ± 0.16	

*BSA* body surface area, *SD* standard deviation

### Association between mean BSA and surgical outcomes

#### Surgical complications

**Fig. 2:**
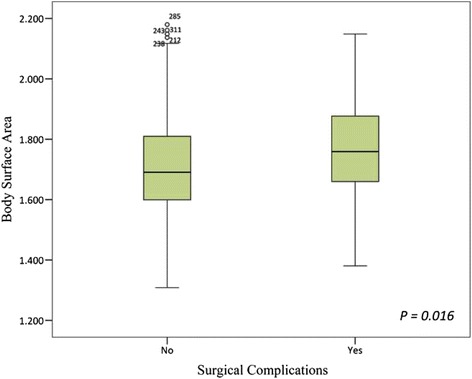
Box-plots revealing the difference in mean body surface area between the patients with surgical complications and patients without surgical complications

The mean BSA values of PSC group and non-PSC group were 1.76 ± 0.15 m^2^ and 1.71 ± 0.16 m^2^, respectively (Table 1; Fig. 2). The mean BSA in PSC group was significantly higher than that in non-PSC group (*P* = 0.016).

#### Overall, pulmonary and cardiovascular complications

As shown in Table 2, no significant difference was discovered in the mean BSA values between the groups classified by overall morbidity (*P* = 0.44), pulmonary complications (*P* = 0.99) and cardiovascular complications (*P* = 0.19).

### ROC analysis on prediction of BSA for PSCs

**Fig. 3:**
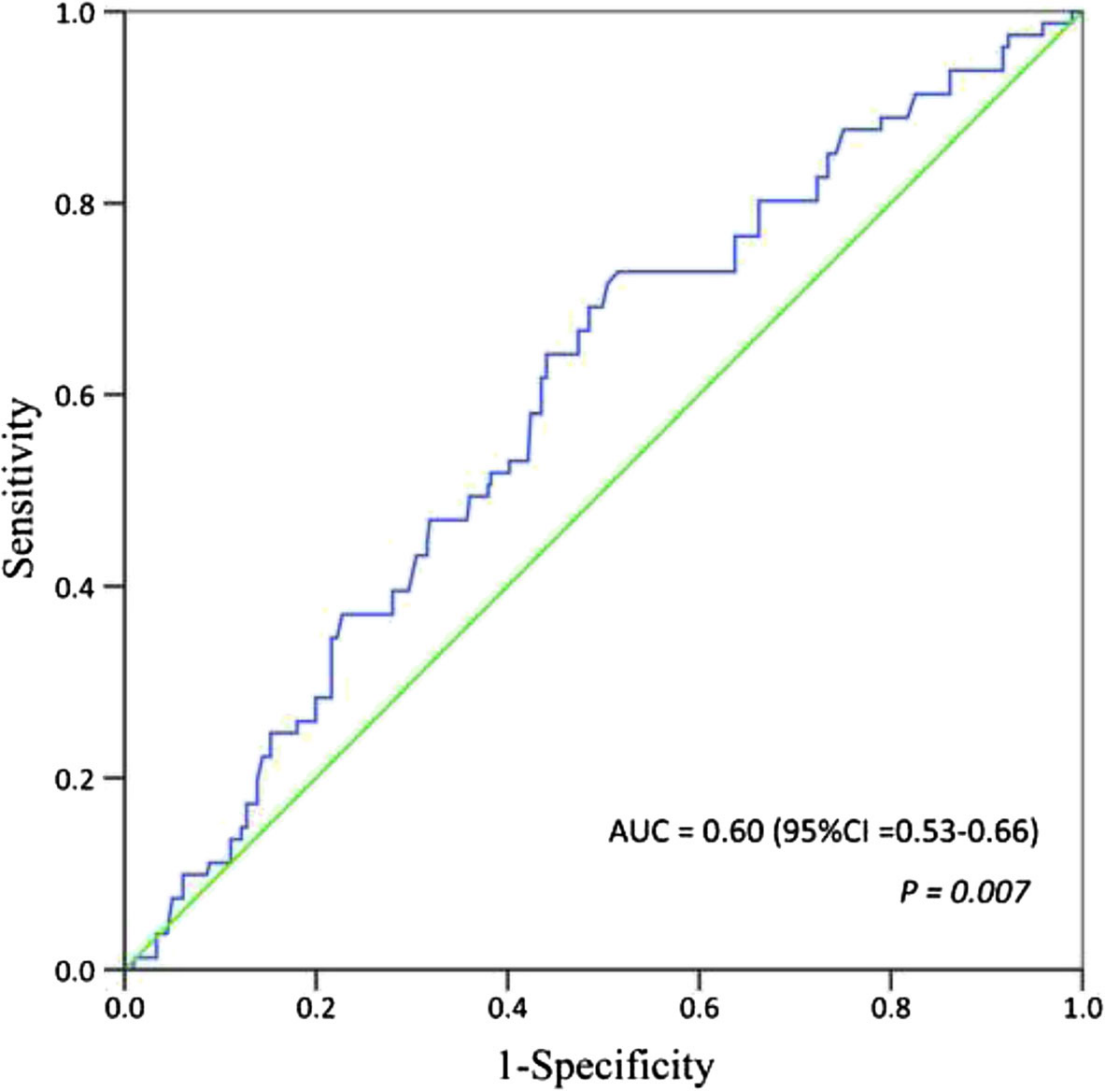
Receiver operating characteristic analysis on the discriminative power of body surface area for predicting postoperative surgical complications. AUC: area under curve; CI: confidence interval

The ROC analysis of BSA showed an AUC of 0.60 (95%CI: 0.53–0.66; *P* = 0.007) on prediction of PSCs (Fig. 3). On the basis of ROC curve, a BSA value of 1.68 m^2^ was found to be the optimal cut-off value with the maximum joint sensitivity of 72.8% and specificity of 48.5%. Therefore, BSA > 1.68 m^2^ was determined as the threshold value for predicting the PSC risk.

### Association between threshold BSA and perioperative parameters

The demographic differences between patients with BSA ≤ 1.68 m^2^ and patients with BSA > 1.68 m^2^ are summarized in Table S2 (see *the* Additional File 1). Compared to patients with BSA ≤ 1.68 m^2^, patients with BSA > 1.68 m^2^ had higher proportions of elderly people, male gender, overweight/obese body shape (BMI > 25.0 kg/m^2^), smoking history, hypertension, adjuvant chemotherapy, lobar resections, lymphatic metastasis and stage II-IIIa cancers. However, the ratio of tumor located in right upper lobe in patients with BSA > 1.68 m^2^ was lower than that in those with BSA ≤ 1.68 m^2^.

### Association between threshold BSA and development of individual PSCs

The incidences of individual PSCs are presented in Table 3. Four most frequent PSCs were PAL (*n* = 52, ratio = 11.8%), subcutaneous emphysema (*n* = 29, ratio = 6.6%), pneumothorax (*n* = 15, ratio = 3.4%) and chylothorax (*n* = 14, ratio = 3.2%).

**Table 3:** Individual surgical complications

Single complication	Total (*N* = 442)	BSA ≤ 1.68 m^2^ (*N* = 197)	BSA > 1.68 m^2^ (*N* = 245)	*P* value
Prolonged air leak (>5 days)	52 (11.8%)	14 (7.1%)	38 (15.5%)	0.006
Subcutaneous emphysema	29 (6.6%)	9 (4.6%)	20 (8.2%)	0.13
Pneumothorax	15 (3.4%)	7 (3.6%)	8 (3.3%)	0.87
Chylothorax	14 (3.2%)	1 (0.5%)	13 (5.3%)	0.004
Wound infection	2 (0.5%)	2 (1.0%)	0 (0.0%)	0.20
Hemothorax	1 (0.2%)	0 (0.0%)	1 (0.4%)	1.0
Bronchial fistula	1 (0.2%)	0 (0.0%)	1 (0.4%)	1.0

*BSA* body surface area

The overall incidence of PSCs in patients with BSA > 1.68 m^2^ was significantly higher than that in those with BSA ≤ 1.68 m^2^ (*n* = 59, ratio = 24.1% vs *n* = 22, ratio = 11.2%; *P* < 0.001). Compared to patients with BSA ≤ 1.68 m^2^, patients with BSA > 1.68 m^2^ had significantly higher incidences of PAL (*n* = 38, ratio = 15.5% vs *n* = 14, ratio = 7.1%; *P* = 0.006) and chylothorax (*n* = 13, ratio = 5.3% vs *n* = 1, ratio = 0.5%; *P* = 0.004). No significant difference was found in the other PSCs between these two groups (Table 3).

### Multivariate analysis on predictors for PSCs

Compared with the non-PSC group, patients in the PSC group had a significantly higher mean age (*P* = 0.044) and proportions of male sex (*P* < 0.001), smoking history (*P* < 0.001), COPD (*P* = 0.005), PRI (*P* < 0.001), hypertension (*P* = 0.005), lobar resection (*P* = 0.011), dense pleural adhesion (*P* = 0.007) and advanced tumor invasion (*P* = 0.030), as shown in Table 1.

Our multivariate logistic-regression model included all the above significant parameters and the threshold value of BSA. Finally, the male sex (OR: 2.98; 95% CI: 1.23–7.23; *P* = 0.016), BSA > 1.68 m^2^ (OR: 2.03; 95% CI: 1.09–3.76; *P* = 0.025), PRI (OR: 3.13; 95% CI: 1.45–6.77; *P* = 0.004) and lobar resection (OR: 2.74; 95% CI: 1.03–7.34; *P* = 0.044) were identified as independent risk factors for PSCs in the multivariate analysis, as shown in Table 4. Higher age (OR: 1.04; 95% CI: 0.99–1.08; *P* = 0.056) and hypertension (OR: 1.65; 95% CI: 0.95–2.86; *P* = 0.074) were both almost predictive of PSCs but failed to reach statistical significance.

**Table 4:** Multivariate analysis on predictors for surgical complications

Estimated factors	Odds ratio	95% confidence interval	*P* value
Age (each 1 year increased)	1.04	0.99–1.08	0.056
Gender (Male vs female)	2.98	1.23–7.23	0.016
Smoking history	1.08	0.53–2.20	0.84
BSA > 1.68 m^2^ vs BSA ≤ 1.68 m^2^	2.03	1.09–3.76	0.025
COPD	1.23	0.66–2.30	0.52
Hypertension	1.65	0.95–2.86	0.074
Preoperative respiratory infection	3.13	1.45–6.77	0.004
Extent of surgery (Lobar vs sub-lobar)	2.74	1.03–7.34	0.044
Dense pleural adhesion	1.56	0.89–2.76	0.12
Tumor invasion (T_3–4_ vs T_1–2_)	2.57	0.87–7.61	0.089

*BSA* body surface area, *COPD* chronic obstructive pulmonary disease

## Discussion

### Key results

The present single-center retrospective study examined the relationships between BSA and development of PSCs in patients undergoing VATS anatomical resections for LAC. We found that the patients with larger BSA had significantly higher incidences of PSCs, especially of PAL and chylothorax. No significant effect of BSA was discovered on the other major postoperative outcomes, including overall morbidity, pulmonary and cardiovascular complications. The ROC analysis showed a threshold BSA value of 1.68m^2^ on prediction of PSC occurrence, with the maximum joint sensitivity of 72.8% and specificity of 48.5%. After further adjusting the risk factors by multivariate logistic-regression analysis, male sex, BSA > 1.68 m^2^, PRI and lobar resection were competent to be independent predictors for surgical complications following VATS anatomical resections for LAC.

### Interpretations

In our study, PSCs occupied the largest proportion of all types of complications, with a rate of 18.3%. PAL was one of minor complications related to surgical procedures and occurred in approximately 8%–15% patients who underwent VATS procedures [19, 20]. In our study, PAL was the most frequent post-VATS complication that were developed in 52 patients, with a rate of 11.8%. This result was similar to the PAL incidences reported in the literature [19, 20]. The prevalence rates of subcutaneous emphysema and pneumothorax in our cohort, followed by the incidence of PAL, were 6.6% and 3.4%, respectively. Almost all of these patients had air leaks that were found from the chest drainage on postoperative day 1, and then persisted beyond the normal hospital stay. That also showed a close relationship of PAL to the risks of pneumothorax and emphysema.

With regard to severe PSCs, chylothorax was developed in 14 patients, followed by bronchial fistula and hemothorax occurred in one patient, respectively. Chylothorax was a relatively rare complication following pulmonary resections that was associated with the mediastinal lymph node dissection and highly variable courses of thoracic duct [10]. The incidence of chylothorax in our cohort was 3.2%, which was within the confines of chylothorax rates ranged 2.1–4.0% in the literature [10, 21, 22]. Bronchial fistula was another one fatal complication, revealing a devastating leakage from airways into the pleural space [9, 23]. The most recent evidence-based reviews had showed a bronchial fistula rate ranged 2.4–3.9% following lung cancer surgery [23–25]. There was only one patient in our cohort developed with a post-lobectomy bronchial fistula, with a rate of 0.2%. We speculated that patients who underwent pneumonectomy were initially excluded before performing this retrospective analysis, and this exclusion criteria led to a large decline in fistula incidence because bronchial fistula occurred more frequently after pneumonectomy than after any other anatomical resection [24].

The present study was the first to show the impact of BSA on the development of surgical complications following VATS anatomical resections for LAC. BSA is a simple way to measure the body size. In general, BSA has a much closer relationship with height than BMI because of essential differences between their calculating formulas [12]. Accordingly, BSA may be more related to the anthropometric measurements and finally affect some major perioperative outcomes, such as the conversion to open surgery and operating time [12]. Several formulas have been proposed to calculate the baseline BSA in different races. In our study, an updated formula appropriate for contemporary Chinese peoples was used to calculate the BSA values [15]. Because of the scarcity of evidence addressing on the association between BSA and VATS outcomes, we explored the predictive value of BSA for PSCs by a ROC analysis to confirm an optimal cut-off value that has the discriminatory ability on prediction of PSC risk. Finally, a BSA value of 1.68m^2^ was determined as the threshold value. Patients with BSA > 1.68 m^2^ had significantly higher PSC incidences than those in patients with BSA ≤ 1.68 m^2^. After minimizing the influence of other parameters by multivariate logistic-regression analysis, BSA > 1.68 m^2^ was found to be a strongly independent predictor of PSCs.

To our knowledge, there was only one retrospective analysis evaluating the effects of BSA on major surgical outcomes in 208 consecutive robotic-assisted lobectomies [13]. The authors demonstrated that patients with larger BSA had a slightly higher morbidity rate than those with smaller BSA, without reaching statistical significance (*P* = 0.28). The incidences of PAL and chylothorax were similar between these two groups, while the incidences of pneumothorax and hemothorax in patients with larger BSA were slightly higher than those in patients with smaller BSA. However, no multivariate analysis was conducted to explore the clinical significance of BSA throughout that study [13]. That was the biggest difference compared to what we analyzed in the present study. Our series showed a significantly higher PSC rate in the patients with larger BSA and further identified that BSA > 1.68 m^2^ could be an independent risk factor for the occurrence of PSC in the multivariate logistic-regression model.

It is understandable that smaller body size can lead to a limited access to the operative field in cardiothoracic surgery. Patients with smaller BSA are considered to have smaller pleural cavities, resulting in the limited visualization and instrument mobility during the VATS procedure. Therefore, as what Frank et al. [13] suggested in their cohort, small body habitus has the probability to increase the surgical difficulty and prolong the operating time, and then cause a higher risk of PSCs. However, our results, which were derived from a larger cohort of patients, were at variance with this explanation. On the contrary, large body size seemed to be more commonly associated with the development of PSCs.

There was no credible evidence to demonstrate this unexpected phenomenon. We speculated that the following three perspectives might be considered for explanations.

Firstly, patients with a larger pleural cavity might have larger area related to severe pleural adhesion and fibro-calcification, which needed to be dissected and divided during the VATS procedure, resulting in a higher probability of air leaks. Secondly, on the basis of similar weight, patients with larger BSA were generally higher than those with smaller BSA. These tall patients with larger BSA might easily suffer from the parenchymal alveolar air leaks induced by damage of visceral pleura on the residual lung when they were encouraged to cough and do weight training after surgery. A tall thin body habitus can predispose to this because of the increased pressure gradient between the lung base and apex, resulting in increased alveolar distending pressures at the apex [26]. Finally, the protective effects of parietal adipose tissues on the surface of lung parenchyma in tall thin patients might be slightly weakened due to their decreased thickness. And that might contribute to the development of alveolar air leaks.

### Generalizability

In the clinical practice, a comprehensive preoperative assessment of surgical risk can affect surgeons’ decision on the surgical procedures. According to our study results, thoracic surgeons can consider to integrate a BSA threshold value into the assessment model to stratify the risk of complications. That may assist to design the best therapeutic strategy for surgical patients, although their BSA could not be modified by preoperative interventions. Furthermore, it may help to select participants in a surgeon’s early learning curve or in a teaching program of VATS techniques.

### Limitations

Several limitations must be taken into account regarding the interpretations.

First, our study was subject to the inherent limitations of any single-center retrospective analysis. The retrospective nature makes it difficult to control the selection bias. Although we have involved all the significant variables into the multivariate logistic-regression model to minimize their bias risks, a propensity-score matched comparison may be more reliable.

Second, our sample size is relatively small. That may cause inadequate comparisons between two groups which are divided by threshold value of BSA, especially in terms of some rare PSCs, resulting in a low evidence power.

Third, the morbidity rate could be also dependent of surgeon’s expertise. However, it might be difficult to perform an appropriate quantitative analysis on this artificial factor. This was another one limitation that could not be ignored.

Fourth, the calculating formula of BSA used in our study was developed based on Chinese subjects. Thus, our findings should be judiciously considered in other ethnic populations.

Fifth, compared with the results reported from similar studies of other surgical specialties [12], our ROC analysis showed both slightly lower sensitivity and specificity for threshold value of BSA on prediction of PSCs, although an AUC with its 95% CI reached statistical significance.

Finally, complete details of laboratorial indexes were not available in all patients.

So their relevant parameters were not evaluated in this study.

## Conclusions

The present study demonstrates that BSA can be an excellent categorical predictor for the risk of PSCs in patients undergoing VATS anatomical resections for LAC. Our results suggest that BSA can be considered when informing patients about surgical risks and selecting cases in the early learning curve. Large-scale and multi-institutional studies are expected to confirm and modify our findings in the future.

## Additional file

Additional file 1: Table S1. Showed the details of definitions for preoperative comorbidities estimated in the current study. **Table S2.** showed the demographic differences between patients with BSA ≤ 1.68 m^2^ and patients with BSA > 1.68 m^2^. (DOCX 20 kb)

## Abbreviation

AUC: Area under curve
BMI: Body mass index
BSA: Body surface area
CI: Confidence interval
COPD: Chronic obstructive pulmonary disease
LAC: Lung adenocarcinoma
NSCLC: Non-small cell lung cancer
OR: Odds ratio
PAL: Prolonged air leak
PRI: Preoperative respiratory infection
PSC: Postoperative surgical complication
ROC: Receiver operating characteristic
SD: Standard deviation
STROBE: Strengthening the Reporting of Observational Studies in Epidemiology
VATS: Video-assisted thoracoscopic surgery
