# Supplementary material for: Body surface area is a novel predictor for surgical complications following video-assisted thoracoscopic surgery for lung adenocarcinoma: a retrospective cohort study
Source: BMC Surg. 2017 Jun 12;17:69. doi: 10.1186/s12893-017-0264-4 (PMC5468978; doi:10.1186/s12893-017-0264-4)
Supplement: Additional file 1: Table S1. — Showed the details of definitions for preoperative comorbidities estimated in the current study. Table S2. showed the demographic differences between patients with BSA ≤ 1.68 m2 and patients with BSA > 1.68 m2. (DOCX 20 kb) [file 12893_2017_264_MOESM1_ESM.docx]

***Additional File 1. Appendix Tables***

Table S1. Definitions for preoperative comorbidities

| **Comorbidity** | **Definition** |
| --- | --- |
| **COPD** | Chronic bronchitis with emphysema; Predicted FEV_1_<70% and FEV_1_/FVC<70%. |
| **Tuberculosis** | Past or current infected people of pulmonary tuberculosis. |
| **Preoperative respiratory infection** | Consists of the obstructive pneumonia, aspiration pneumonia, bronchiectasis, lung abscess and bacterial/fungal infection. |
| **Hypertension** | Systolic pressure ≥140mmHg and diastolic pressure ≥90mmHg. |
| **Diabetes mellitus** | Fasting plasma glucose ≥7.0mmol/L; Plasma glucose of two hours after meal ≥11.1mmol/L. |
| **Coronary heart disease** | Previous angina pectoris, coronary atherosclerosis and myocardial infarction (>90days) treated by percutaneous coronary intervention or surgery or medical care. |
| **Renal insufficiency** | Renal dysfunction treated by dialysis. |
| **Severe liver diseases** | Involved the hepatitis B, hepatitis C, Severe fatty liver, hepatocirrhosis and hepatic parasitosis. |
| **Previous malignancy** | Malignancies treated within the previous five years other than the lung cancer being resected. |
| **Steroid use** | A long-term use of corticosteroids (>six months). |

COPD, chronic obstructive pulmonary disease; FEV_1_, forced expiratory volume in one second; FVC, forced vital capacity

Table S2. Comparisons between patients with BSA≤1.68m^2^ and patients with BSA>1.68m^2^

| **Characteristics** | **Total (*N*=442)** | **BSA≤1.68m^2^ (*N*=197)** | **BSA>1.68m^2^ (*N*=245)** | ***P* value** |
| --- | --- | --- | --- | --- |
| ***Basic information*** | | | | |
| Mean age, years (SD) | 62.84±8.07 | 64.11±8.17 | 61.82±7.85 | 0.003 |
| Age categorization | | | | |
| Age ≤65 years | 277 (62.7%) | 109 (55.3%) | 168 (68.6%) | 0.004 |
| Age >65 years | 165 (37.3%) | 88 (44.7%) | 77 (31.4%) |  |
| Gender (Male, %) | 232 (52.5%) | 35 (17.8%) | 197 (80.4%) | <0.001 |
| Mean BMI, kg/m^2^ (SD) | 23.45±3.01 | 22.05±2.52 | 24.57±2.90 | <0.001 |
| BMI categorization | | | | |
| < 18.5 kg/m^2^ | 21 (4.8%) | 18 (9.1%) | 3 (1.2%) | <0.001 |
| 18.5 to 25.0 kg/m^2^ | 300 (67.9%) | 161 (81.7%) | 139 (56.7%) |  |
| > 25.0 to < 30.0 kg/m^2^ | 114 (25.8%) | 18 (9.1%) | 96 (39.2%) |  |
| ≥ 30 kg/m^2^ | 7 (1.6%) | 0 (0.0%) | 7 (2.9%) |  |
| Smoking history | 167 (37.8%) | 29 (14.7%) | 138 (56.3%) | <0.001 |
| ***Preoperative comorbidities*** | | | | |
| COPD | 87 (19.7%) | 32 (16.2%) | 55 (22.4%) | 0.10 |
| Tuberculosis | 44 (10.0%) | 23 (11.7%) | 21 (8.6%) | 0.28 |
| Preoperative respiratory infection | 38 (8.6%) | 16 (8.1%) | 22 (9.0%) | 0.75 |
| Hypertension | 148 (33.5%) | 55 (27.9%) | 93 (38.0%) | 0.026 |
| Diabetes mellitus | 47 (10.6%) | 18 (9.1%) | 29 (11.8%) | 0.36 |
| Coronary heart disease | 45 (10.2%) | 17 (8.6%) | 28 (11.4%) | 0.33 |
| Renal insufficiency | 32 (7.2%) | 12 (6.1%) | 20 (8.2%) | 0.40 |
| Severe liver diseases | 53 (12.0%) | 17 (8.6%) | 36 (14.7%) | 0.056 |
| Previous malignancy | 32 (7.2%) | 15 (7.6%) | 17 (6.9%) | 0.79 |
| Steroid use | 23 (5.2%) | 10 (5.1%) | 13 (5.3%) | 0.91 |
| ***Combined treatment modalities*** | | | | |
| Neoadjuvant therapy | 31 (7.0%) | 10 (5.1%) | 21 (8.6%) | 0.15 |
| Adjuvant chemotherapy | 122 (27.6%) | 35 (17.8%) | 87 (35.5%) | <0.001 |
| ***Intraoperative parameters*** | | | | |
| Tumor location | | | | |
| Right upper lobe | 159 (36.0%) | 81 (41.1%) | 78 (31.8%) | 0.004 |
| Left upper lobe | 100 (22.6%) | 50 (25.4%) | 50 (20.4%) |  |
| Right lower lobe | 78 (17.6%) | 34 (17.3%) | 44 (18.0%) |  |
| Left lower lobe | 61 (13.8%) | 23 (11.7%) | 38 (15.5%) |  |
| Right middle lobe | 44 (10.0%) | 9 (4.6%) | 35 (14.3%) |  |
| Extent of surgery | | | | |
| Single lobectomy | 369 (83.5%) | 155 (78.7%) | 214 (87.3%) | 0.003 |
| Bi-lobectomy | 4 (0.9%) | 0 (0.0%) | 4 (1.6%) |  |
| Segmentectomy | 69 (15.6%) | 42 (21.3%) | 27 (11.0%) |  |
| Pleural invasion | | | | |
| None | 206 (46.6%) | 98 (49.7%) | 108 (44.1%) | 0.49 |
| Visceral | 207 (46.8%) | 87 (44.2%) | 120 (49.0%) |  |
| Parietal | 29 (6.6%) | 12 (6.1%) | 17 (6.9%) |  |
| Pleural adhesion | | | | |
| None | 199 (45.0%) | 89 (45.2%) | 110 (44.9%) | 0.97 |
| Light | 132 (29.9%) | 57 (28.9%) | 75 (30.6%) |  |
| Moderate | 71 (16.1%) | 32 (16.2%) | 39 (15.9%) |  |
| Severe/atresia | 40 (9.0%) | 19 (9.6%) | 21 (8.6%) |  |
| Pulmonary fissure status | | | | |
| Complete fissures | 300 (67.9%) | 131 (66.5%) | 169 (69.0%) | 0.58 |
| Incomplete fissures | 142 (32.1%) | 66 (33.5%) | 76 (31.0%) |  |
| ***Pathological parameters*** | | | | |
| Differentiation degree | | | | |
| Low | 41 (9.3%) | 14 (7.1%) | 27 (11.0%) | 0.16 |
| Moderate/high | 401 (90.7%) | 183 (92.9%) | 218 (89.0%) |  |
| Tumor invasion (T status) | | | | |
| T_1-2_ | 425 (96.2%) | 193 (98.0%) | 232 (94.7%) | 0.075 |
| T_3-4_ | 17 (3.8%) | 4 (2.0%) | 13 (5.3%) |  |
| Lymph node metastasis (N status) | | | | |
| N_1-2_ | 78 (17.6%) | 15 (7.6%) | 63 (25.7%) | <0.001 |
| N_0_ | 364 (82.4%) | 182 (92.4%) | 182 (74.3%) |  |
| TNM stage | | | | |
| I | 346 (78.3%) | 171 (86.8%) | 175 (71.4%) | <0.001 |
| II | 54 (12.3%) | 20 (10.1%) | 34 (13.9%) |  |
| IIIa | 42 (9.5%) | 6 (3.0%) | 36 (14.7%) |  |

BMI, body mass index; BSA, body surface area; COPD, chronic obstructive pulmonary disease; SD, standard deviation
